# Eltrombopag Improves Refractory Thrombocytopenia in a Patient with Systemic Lupus Erythematosus

**DOI:** 10.1155/2018/6305356

**Published:** 2018-08-15

**Authors:** Natsuki Shima, Keiichi Sumida, Masahiro Kawada, Akinari Sekine, Masayuki Yamanouchi, Rikako Hiramatsu, Noriko Hayami, Eiko Hasegawa, Tatsuya Suwabe, Junichi Hoshino, Naoki Sawa, Kenmei Takaichi, Kenichi Ohashi, Takeshi Fujii, Yoshifumi Ubara

**Affiliations:** ^1^Nephrology Center, Toranomon Hospital, Kawasaki, Kanagawa, Japan; ^2^Okinaka Memorial Institute for Medical Research, Toranomon Hospital, Tokyo, Japan; ^3^Department of Pathology, Toranomon Hospital, Tokyo, Japan; ^4^Department of Pathology, Yokohama City University Hospital Graduate School of Medicine, Kanagawa, Japan

## Abstract

A 42-year-old woman with systemic lupus erythematosus (SLE) was admitted to our hospital for evaluation of severe thrombocytopenia. She was treated with steroids, intravenous cyclophosphamide, intravenous immunoglobulin, and plasma exchange, but her thrombocytopenia did not improve. Renal biopsy showed class IV-S(C) + V lupus nephritis, according to the classification of the International Society of Nephrology/Renal Pathology Society. The PA-IgG and serum thrombopoietin (TPO) levels were elevated. Her thrombocytopenia responded to off-label administration of eltrombopag, which was discontinued after 42 months. At 18 months after stopping eltrombopag, the platelet count was 19.3 × 10^4^/*μ*L. Eltrombopag may be a therapeutic option for SLE patients with severe thrombocytopenia refractory to conventional therapy.

## 1. Introduction

Thrombocytopenia is a frequent hematological manifestation in patients with systemic lupus erythematosus (SLE). Mechanisms causing thrombocytopenia have been suggested, which are increased platelet destruction and impaired platelet production by megakaryocytes in the bone marrow mediated by autoantibodies in the peripheral circulation. SLE-associated thrombocytopenia is usually treated with glucocorticoids, immunosuppressants such as azathioprine and cyclophosphamide, intravenous immunoglobulin (IVIG), or splenectomy. If thrombocytopenia shows a poor response to these conventional therapies, monoclonal antibodies targeting the thrombopoietin (TPO) receptor have recently become another treatment option [1]. Eltrombopag is a TPO receptor agonist that has been approved for patients with chronic idiopathic (immune) thrombocytopenic purpura (ITP) showing an insufficient response to conventional therapy [2]. It also has the potential to be effective for refractory thrombocytopenia in patients with SLE, as demonstrated by the following case report.

## 2. Case Report

**Figure 1:**
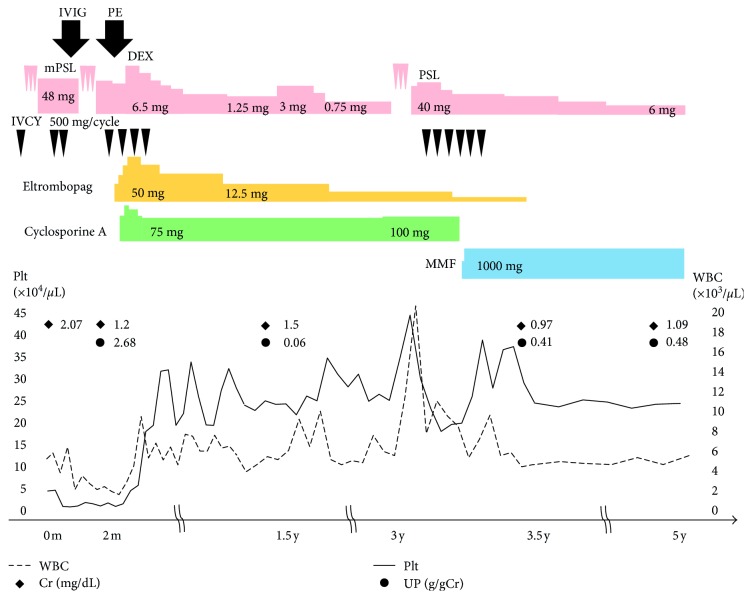
Clinical course. 0 m = 1 month before admission. IVIG: intravenous immunoglobulin; PE: plasma exchange; DEX: dexamethasone; IVCY: intravenous cyclophosphamide; UP: urinary protein.

A 42-year-old Japanese woman was admitted to our hospital for evaluation of severe thrombocytopenia. At the age of 34, SLE was diagnosed on the basis of a malar rash, oral ulcers, arthritis, and positivity for anti-double-stranded- (ds-) DNA antibody. Prednisolone was started at a dose of 30 mg/day, after which tacrolimus was added at 3 mg daily. Her SLE improved, and prednisolone was tapered to 3 mg/day. One month before admission, proteinuria increased to 2.34 g daily. Serum albumin was 2.0 g/dL (normal: 3.9–5.2), and creatinine was 2.3 mg/dL (normal: 0.46–0.78). Anti-ds-DNA antibody showed an increase to 90 IU/mL (normal: <12). The platelet count was decreased to 4.4 × 10^4^/*μ*L, and CH50 was 5 U/mL (normal: >30 U/mL) (Table 1). Antiplatelet drugs or any other drugs that could adversely affect platelet numbers were not taken. Intravenous methylprednisolone pulse therapy (1000 mg/day for three days) was initiated, followed by methylprednisolone at 48 mg/day. She also received five monthly cycles of intravenous cyclophosphamide (IVCY) pulse therapy (500 mg per cycle). Although her renal function, complement levels, and the anti-ds-DNA antibody titer all improved, thrombocytopenia became worse and the patient was admitted to our hospital (Figure 1).

On admission, she had diffuse purpura on the anterior chest and limbs. The lungs were clear on auscultation. The liver and spleen were not palpable, and there was no evidence of arthritis or pathological bleeding such as epistaxis. Pitting edema of both legs was noted.

Laboratory findings were as follows (Table 1): the white blood cell count was 6,300/*μ*L (86.5% neutrophils and 7.5% lymphocytes), hemoglobin was 8.7 g/dL, reticulocyte count was 9.3 × 10^4^/*μ*L (3.5%), and platelet count was 0.8 × 10^4^/*μ*L (10.5% immature platelets). The direct Coombs test was negative. Anemia secondary to chronic inflammation caused by SLE was considered. In addition, serum albumin was 3.1 g/dL, blood urea nitrogen was 33 mg/dL (normal: 8–21), creatinine was 1.2 mg/dL, and eGFR was 38.8 ml/min/1.73 m2. C-reactive protein was 0.0 mg/dL. Antinuclear antibody (ANA) was positive at 1:40 with a speckled pattern, while anti-ds-DNA antibody was elevated to 18 U/mL (normal: <12). The lupus anticoagulant assay was consistently negative, and anticardiolipin antibodies were not detected throughout the clinical course. ADAMTS13 activity was 87% (normal: >10%), and inhibitors were not detected. Total urinary protein excretion was 1.55 g/day. The urine sediment contained 6–10 erythrocytes per high-power field (HPF). Bone marrow examination showed normocellular marrow, and megakaryocyte number was within normal range with 12 (normal range: 10 to 50) number/mm^2^. There was no hepatosplenomegaly on computed tomography of the abdomen.

## 3. Clinical Course

The patient's platelet-associated IgG (PA-IgG) level was elevated to 124.0 ng/10^7^ cells (normal: <46). Although intravenous immunoglobulin (IVIG) was administered and plasma exchange (PEx) was performed, severe thrombocytopenia persisted. Even after the platelet count elevated up to 10.0 × 10^4^/*μ*L after platelet transfusion of 10 units (200 mL) including more than 2.0 × 10^11^ numbers, blood sampling after 1 day showed that the platelet count dropped to 1.0 × 10^4^/*μ*L. Platelet transfusion was repeated. This indicated that immune-mediated thrombocytopenia might contribute to a mechanism of this patient's thrombocytopenia. The serum TPO level was 2.59 FmoL/mL (normal: <1.0). Therefore, off-label treatment with eltrombopag was started at a dose of 12.5 mg orally and increased up to 50 mg daily. The platelet count increased to 31.6 × 10^4^/*µ*L and more than twice the baseline. Eltrombopag was discontinued after 42 months, but the platelet count was 19.3 × 10^4^/*μ*L at 18 months after discontinuation. She developed lupus enteritis about three years after the hospital admission, but there was no relapse of thrombocytopenia (Figure 1). Cyclosporine A and mycophenolate mofetil were used for induction and maintenance therapy of lupus nephritis.

## 4. Renal Biopsy Findings

**Figure 2:**
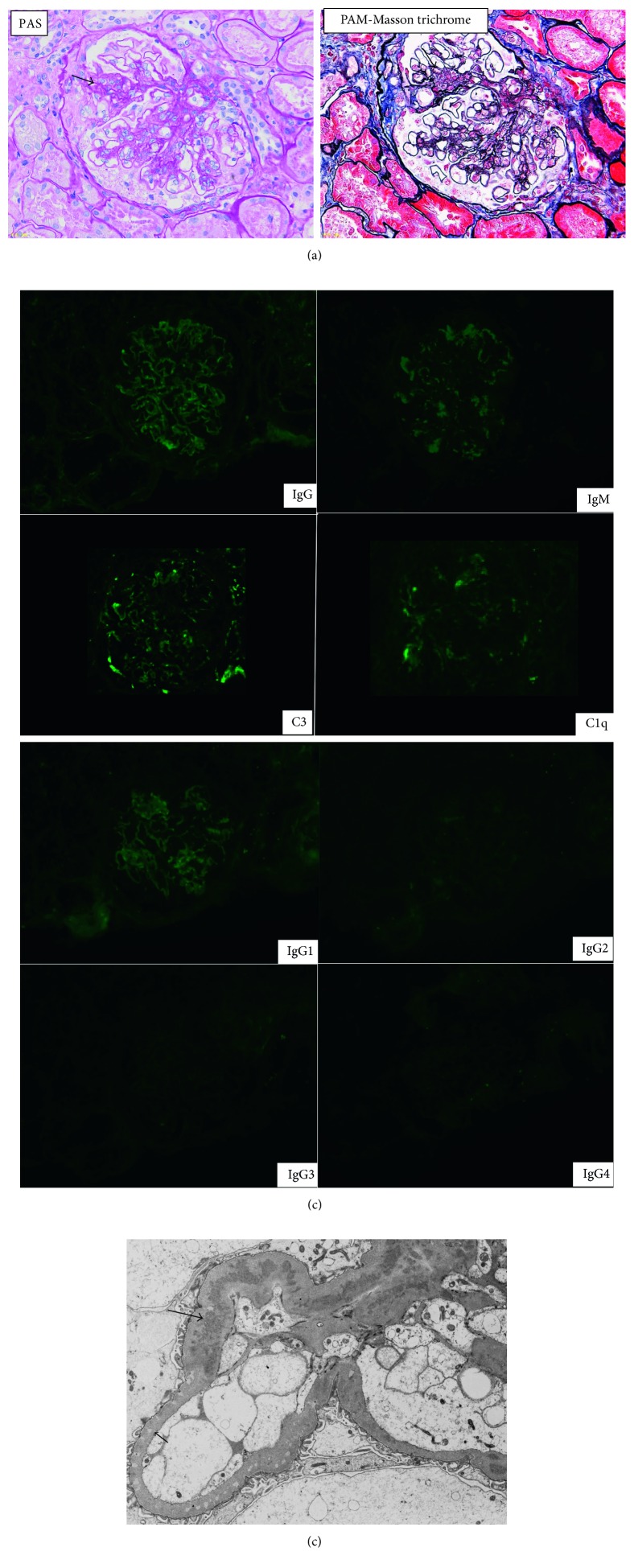
Microscopy of a renal biopsy specimen. (a) There are mainly chronic inactive lesions with scarring, though endocapillary glomerulonephritis is partially noted (arrow). Diffuse thickening of glomerular capillaries is seen. (b) Immunofluorescence is weakly positive for granular deposits of IgG, IgM, C3, and C1q along the GBM. Analysis of IgG subclasses revealed deposition of IgG1. (c) Electron microscopy shows subepithelial and intramembranous electron-dense deposits (EDD; small arrow) and electron-lucent deposits (large arrow), along with partial mesangial and subendothelial EDD.

Light microscopic (LM) examination of a renal biopsy specimen revealed global sclerosis in 8 out of 41 glomeruli. More than 50% of the glomeruli displayed segmental lesions. While chronic inactive lesions with scarring were prominent, endocapillary glomerulonephritis was noted partially. Diffuse thickening of glomerular capillaries was seen (Figure 2(a)). Immunofluorescence (IF) was weakly positive for granular deposits of IgG, IgM, C3, and C1q along the glomerular basement membrane (Figure 2(b)). Analysis of IgG subclasses showed that IgG1 was predominantly positive. On electron microscopy (EM), subepithelial and intramembranous electron-dense deposits (EDD) and electron-lucent deposits were mainly noted, but mesangial and subendothelial EDD were also partially detected (Figure 2(c)). Class IV-S (C) + V lupus nephritis was diagnosed according to the classification of the International Society of Nephrology/Renal Pathology Society, with the above findings representing typical renal histology after immunosuppressant therapy including steroids.

## 5. Discussion

Many patients with ITP or secondary ITP due to lymphoproliferative disorders responding to eltrombopag have already been reported, but the efficacy of this drug for thrombocytopenia associated with SLE remains unclear, and only three reports have been published [3–5]. Maroun et al. reported 3 SLE patients with thrombocytopenia refractory to glucocorticoids, in whom elevation of the platelet count was achieved by administration of eltrombopag. In one patient, the platelet count remained above 350,000/mm^3^ for six months after discontinuation of the drug [3]. Scheinberg et al. reported a 30-year-old woman with SLE and severe thrombocytopenia refractory to conventional therapy. Administration of eltrombopag was initially effective, but was discontinued. After one week, her platelet count decreased to 2,000/*µ*L, so eltrombopag was restarted and the platelet count increased to 150,000/*µ*L [4]. Magnano et al. reported a 69-year-old woman who had SLE and severe thrombocytopenia refractory to both IVIG and rituximab. Treatment with eltrombopag (25 mg/day) was started, and a complete response (platelet count > 100,000/mm^3^) was achieved after 2 weeks, which was maintained after 5 months [5]. No adverse effects of eltrombopag were reported in these patients.

Eltrombopag binds to the transmembrane portion of the TPO receptor (c-MPL) and stimulates the early to late stages of megakaryocyte maturation, resulting in production of platelets. Eltrombopag also stimulates trilineage proliferation of hematopoietic stem cells [6], and it has been reported to be effective for aplastic anemia refractory to immunosuppression, improving the hemoglobin level and neutrophil count as well as the platelet count [7].

Romiplostim is another thrombopoietin receptor agonist which is administered intravenously. IL-11 could be considered if the patient does not respond adequately to conventional therapies.

Hydroxychloroquine would be regarded as a standard baseline immunosuppressant in SLE, but it was not authorized in Japan at that time. The reason why tacrolimus was added has not been known because it had been added at the other hospital.

The biggest difference between SLE and isolated ITP on the management is therapeutic indications. Glucocorticoid pulse therapy may be quickly introduced despite platelet count in SLE-associated ITP which can be concomitant with severe organ involvement such as neuropsychiatric syndrome or diffuse alveolar hemorrhage. However, mild thrombocytopenia in patients with isolated ITP requires no specific therapy other than regular monitoring. If we correctly assess organ involvement of SLE, patients can cause less adverse effects of corticosteroids such as infection.

In conclusion, we encountered a female SLE patient with thrombocytopenia refractory to conventional therapy and elevation of the serum TPO level. Off-label use of eltrombopag was effective for increasing her platelet count, which was 19.3 × 10^4^/*μ*L even 18 months after this drug was discontinued. Although autoantibody-mediated destruction of platelets in the peripheral circulation has been considered the chief mechanism of SLE-related thrombocytopenia, impaired platelet production by megakaryocytes in the bone marrow mediated by autoantibodies might be more important in some patients. Conventional therapies are effective for the former mechanism, while eltrombopag may be a useful option for the latter mechanism.

## Figures and Tables

**Table 1:** Laboratory data.

	One month before admission	On admission	Before start of eltrombopag	Five years after admission	Normal range
White blood cell (/*µ*L)	5,000	6,300	2,000	5,500	3,200–7,900
Red blood cell (×10^6^/*µ*L)	9.2	8.7	8.2	11.0	11.3–15.0
Platelet count (×10^4^/*µ*L)	4.4	0.8	1.0	21.6	15.5–35.0
Total protein (g/dL)	5.0	6.5	6.0	5.7	6.9–8.4
Albumin (g/dL)	2.0	3.1	3.2	3.7	3.9–5.2
Urea nitrogen (mg/dL)	48.5	33	28	22	8–21
Creatinine (mg/dL)	2.07	1.2	1.2	1.09	0.46–0.78
eGFR (mL/min/1.73 m^2^)	22.1	38.8	42.1	43.2	—
IgG (mg/dL)	1,206	1,603	1,010	574	870–1700
C3 (mg/dL)	23	43	58	78	86–160
C4 (mg/dL)	3.5	9	17	28	17–45
CH50 (U/mL)	5	21	27	43	30–50
Anti-ds-DNA antibody (IU/mL)	90.7	18	12	67.9	<10.0
Urinary RBC sediment (/HPF)	50–99	6–10	11–30	<1	<1
Urinary protein (g/gCr)	2.34	1.55	2.68	0.48	—

eGFR: estimated glomerular filtration rate; ds-DNA: double-stranded-DNA.

## Abbreviations

SLE: Systemic lupus erythematosus
IVIG: Intravenous immunoglobulin
TPO: Thrombopoietin
IVCY: Intravenous cyclophosphamide
ANA: Antinuclear antibody
eGFR: Estimated glomerular filtration rate
HPF: High-power field
LM: Light microscopy
IF: Immunofluorescence
EM: Electron microscopy
ISN/RPS: International Society of Nephrology/Renal Pathology Society classification
ITP: Idiopathic (immune) thrombocytopenic purpura
PAM: Periodic acid-methenamine-silver.
